# Advocacy for family medicine in sub-Saharan Africa

**DOI:** 10.4102/phcfm.v17i1.5109

**Published:** 2025-08-12

**Authors:** Robert J. Mash

**Affiliations:** 1 Division of Family Medicine and Primary Care, Faculty of Medicine and Health Sciences, Stellenbosch University, Cape Town, South Africa

Family medicine should ‘stop talking to ourselves’ and boldly present its ‘value proposition’ to policymakers in the African region. This is the advice of Prof. Nelson Sewankambo at the Primary Care and Family Medicine (PRIMAFAMED) network meeting in Lusaka, June 2025. The *African Journal of Primary Health Care and Family Medicine* recently published a special collection to reflect on the lessons learnt regarding advocacy for family medicine in sub-Saharan Africa.

Table 1 illustrates the need for bold and energetic advocacy across the region. Family physicians from 11 African countries have established the East, Central and Southern African College of Family Physicians (ECSA-CFP) with the goal of increasing the supply of family physicians. The current number of family physicians in these countries ranges from 0/10 000 population in Zimbabwe and Tanzania to 0.2/10 000 population in Botswana. This can be compared to 7–12/10 000 in Europe or 3.4/10 000 in the United States. Training output currently ranges from less than 5 graduates per year to a maximum of 20 per year. The need to scale-up the supply and deployment of family physicians is clear.

**TABLE 1:** Employment and training of family physicians in 11 African countries linked to the East, Central and Southern African College of Family Physicians.

Country	Doctors /10 000 population	FPs / 10 000 population	Population (millions)	Employment of family physicians within the health system	Postgraduate education (number of graduates per year)
Angola	2.4	0.01	37	Training and deployment of family physicians in PHC went to scale with government support in 2019.	National Programme for the Expansion of Family Medicine with support from Cuba (400)
Botswana	3.8	0.19	2	Policy is to deploy family physicians at primary hospitals and to support PHC.	One training programme with three training complexes (one urban and two rural) (< 10)
DR Congo	1.9	0.01	106	Family medicine was recognised as a speciality in 2021 but no policy to deploy family physicians.	One training programme is active out of 57 medical schools (< 5)
Ethiopia	1.1	0.01	123	Strong policy on strengthening PHC and the human resources, including family physicians.	One training programme developed in collaboration with University of Toronto, Canada (20)
Kenya	1.0	0.03	55	Policy is to deploy family physicians in each county across the country. Strong government commitment to strengthen PHC and deployment of family physicians.	Five training programmes; three in the public sector and Aga Khan and Kabarak in the private sector, two urban and three rural settings. Moi and Aga Khan are the stronger programmes (< 20)
Lesotho	1.5	0.05	2	There is government commitment to train family physicians, but graduates are just emerging.	Collaboration between government ministry of health and University of Boston to train family physicians (< 5)
Malawi	0.5	0.01	21	Strong policy commitment to strengthen PHC and family physicians are being deployed.	One training programme (< 10)
Tanzania	0.4	0.00	67	Policy on family physicians is still being discussed and currently family physicians only in the private sector.	One training programme that serves the primary care facilities operated by Aga Khan (< 5)
Uganda	1.7	0.02	49	Policy is to deploy family physicians in each district across the country and to strengthen PHC.	Two training programmes, although Makerere is the stronger (< 10)
Zambia	2.6	0.01	21	Family physician training has just started, and graduates are only just emerging, no clear policy.	One training programme. The first graduate is running the programme (< 5)
Zimbabwe	1.7	0.00	16	Family physician training has just started, and graduates are only just emerging, no clear policy.	Two training programmes. The one has no family physicians on faculty. Supported by Stellenbosch University and Academics Without Borders (< 5)

Note: Information derived from https://worldpopulationreview.com/ and members of ECSA-CFP.

PHC, primary health care.

The scale-up of family medicine, however, will depend on the value proposition and their cost-effective contribution to the health systems in their respective countries. The evidence for the value of family physicians in African health systems continues to grow, although it is limited by the small numbers in many countries.^1,2^ Countries with larger numbers have a responsibility to generate the evidence. Some countries have published position articles that state the value proposition.^3^ For example, in South Africa the family physician is described as a clinician and consultant to healthcare teams in primary care and primary hospitals. They capacitate these healthcare teams and also train undergraduate or postgraduate students and interns. They lead these teams and implement clinical governance activities to improve the quality of care, patient safety and resilience of facilities and services.

As family physicians fulfil their roles in primary hospitals and primary care, they strengthen the part of the health system that is most able to improve health status and equity. They enable people to obtain care for more complex conditions close to their homes, avoid the need for referral and provide effective follow up on discharge of patients with complicated problems.^4^ Their contribution is often mentioned in the context of expanding surgical capacity, reducing litigation around maternal and neonatal care, improving care for non-communicable diseases and children. More recently their contribution to implementing palliative care services has also been highlighted.^5,6^

There are many misconceptions or myths about family physicians among policymakers and other stakeholders.^7^ For example:

- ‘Newly qualified doctors are general practitioners’.
  The implication is that no further postgraduate training is needed to work as a competent family physician. In their 2024 Primary Health Care Primer, the World Health Organization (WHO) states that ‘long mistakenly associated with the absence of “special skills” generalist medicine is increasingly recognised as requiring purposeful training. In many countries, highly trained generalist physicians responsible for high-quality primary care (and sometimes secondary care) and trained according to the patient-centred clinical method, are called family physicians’.
- ‘We want to replace nurse practitioners and clinical officers with family physicians’.
  The proposed model of care is not to make family physicians the first contact primary care provider, but to include access to a family physician in primary care teams so that the comprehensiveness and quality of care can be improved.
- ‘Family physicians are for private practice’.
  Family physicians in private practice work similarly to their counterparts in high income countries. However, if we want to make a difference to populations in Africa the family physician must be incorporated into the public sector model of care.

We need to advocate better and communicate the value proposition more clearly. Michael Kidd, recently appointed as the Chief Medical Officer for Australia, and a family physician, has outlined seven key principles for effective advocacy:^8^

- Understand the issue: We must not only be experts in our discipline and the value proposition but also understand the policy landscape and how we can contribute to the priorities of policymakers in our context. It is about our contribution and not our status.
- Identify the right audience: Identify the policymakers and stakeholders in your setting and know the key people. Universities and higher education bodies are key to including family medicine in undergraduate education and enabling postgraduate training. The Ministry of Health and regulatory bodies are also critical. It is helpful to speak on behalf of a national professional body such as an academy or college.^7^ If you do not have a professional association in your country, then a good starting point would be to create one, even if you are just a few people. This would also enable you to participate in organisations such as WONCA (World Organization of Family Doctors) and ECSA-CFP.
- Build relationships: Find ways of engaging key people and building strategic partnerships.^7^ Invite people to speak at a workshop, seminar or conference, or to write something for a local journal. You may then be invited to participate in advisory bodies or task teams and to offer leadership in your area of expertise.
- Use evidence and data: We need to know the evidence from our context and to take every opportunity to create new evidence through research that investigates our contribution and value. I think we also need to engage in economic analysis that evaluates the cost of going to scale and return on investment. For example, I estimate the cost of going to scale in South Africa would be around R1 billion to fund the posts. In the national frame this is not unfeasible as one tertiary hospital costs R3-4 billion a year and we spend R20 billion a month on social grants.
- Craft a clear message: Be ready with your ‘elevator pitch’. If you have 5 min with a key stakeholder, what would you say? Publishing a national position article can also help reach consensus on a clear message and articulation of the value of family physicians.
- Engage the public: Most public do not know what a family physician is in the African context. We need to become comfortable at using social and traditional media to communicate who we are and what value we bring.
- Use the media: Beyond scientific journals there is a wide variety of media that can be used and tailored to your audience and message.^7^ For example, opinion editorials, policy briefs, blogs, videos, podcasts, and social media posts.

Advocacy can take place at multiple levels, and everyone can play a part.^7^ At a regional level, organisations such as WONCA and PRIMAFAMED can engage with WHO Afro and other regional entities. African midwives are also organising a new regional professional body to enable advocacy.^9^ In-country national professional bodies should engage with ministries, regulatory bodies and higher education institutions. At a sub-national level, academics and senior clinicians can engage at the provincial or state level. Even at the district level individual family physicians can explain and advocate for the role of the family physician.
